# Computational study of the blood hemodynamic inside the cerebral double dome aneurysm filling with endovascular coiling

**DOI:** 10.1038/s41598-023-29988-w

**Published:** 2023-02-19

**Authors:** Ali Rostamian, Keivan Fallah, Yasser Rostamiyan, Javad Alinejad

**Affiliations:** Department of Mechanical Engineering, Sari Branch, Islamic Azad University, Sari, Iran

**Keywords:** Biomedical engineering, Mechanical engineering

## Abstract

The rupture of the aneurysm wall is highly associated with the hemodynamic feature of bloodstream as well as the geometrical feature of the aneurysm. Coiling is known as the most conventional technique for the treatment of intracranial cerebral aneurysms (ICA) in which blood stream is obstructed from entering the sac of the aneurysm. In this study, comprehensive efforts are done to disclose the impacts of the coiling technique on the aneurysm progress and risk of rupture. The computational fluid dynamic method is used for the analysis of the blood hemodynamics in the specific ICA. The impacts of the pulsatile blood stream on the high-risk region are also explained. Wall shear Stress (WSS) and Oscillatory shear index (OSI) factors are also compared in different blood viscosities and coiling conditions. According to our study, the hematocrit test (Hct) effect is evident (25% reduction in maximum WSS) in the two first stages (maximum acceleration and peak systolic). Our findings present that reduction of porosity from 0.89 to 0.79 would decrease maximum WSS by about 8% in both HCT conditions.

## Introduction

The formation and progress of several cardiovascular diseases are mainly related to the geometrical factors and hemodynamics. The effect of flow structure and wall shear stress on the creation and development of the atherosclerotic panel which is the main reason for stroke is confirmed according to the available resources^1–3^. Meanwhile, vessel bifurcation may occur in the region where blood stream is severely distributed or stagnant and previous reports confirm that chance of atheroma build-up nearby this section^4,5^. In addition, it was found that the mechanism of plaque production and rupture is controlled by the wall shear stress (WSS). These findings have motivated scholars to investigate the role of WSS in cardiovascular arteries. Among different cardiovascular diseases, a cerebral aneurysm is a crucial disorder that has critical effects on human life^6–8^. Several biomedical researches have been conducted in biology science^9,10^.

Among different aneurysm types, internal carotid artery (ICA) is more conventional in patients^11,12^. The creation and growth of the ICA highly rely on the hemodynamic characteristics of the blood stream and the distribution of the wall shear stress on the artery. In fact, the formation of the sac in the branching of the artery is known as the main outcome of abnormal wall stress^13,14^. Therefore, the precise estimation of the blood hemodynamics could present significant information on the treatment of this disease. Besides, the geometric parameters of aneurysms i.e. location and size have a great influence on the rupture of aneurysms^15,16^. The main challenge for the investigation of cardiovascular disease is the accessible data for the measurement of the blood characteristics i.e. WSS in vivo. Surgeons have extensively used magnetic resonance imaging (MRI) for the measurement of the velocity in these organs. However, the estimation of the WSS as the main factor for the production and rupture of the vessels is not a simple task^17–19^. In fact, tissue and blood velocity in specific sections are available via Phase contrast magnetic resonance imaging (PC-MRI). Nonetheless, accurate prediction of the WSS is obstructed due to low longitudinal in-plane resolution of the image and difficult detection near the artery wall^20,21^. To overcome this problem, the computational fluid dynamic is used to calculate the WSS via simulation of the blood flow in the selected model. Successful simulation of the blood stream requires earful considerations for the modeling of this problem^22–24^. High-precision anatomic models, application of a suitable viscosity model, the accurate boundary conditions for the blood flow, and fluid–solid interactions on the wall of the artery are some of the main considerations for the computational study of the blood flow within the aneurysm. Due to the importance of this topic, there are several models for the real estimation of the blood stream inside the cardiovascular arteries^25,26^.

**Figure 1:**
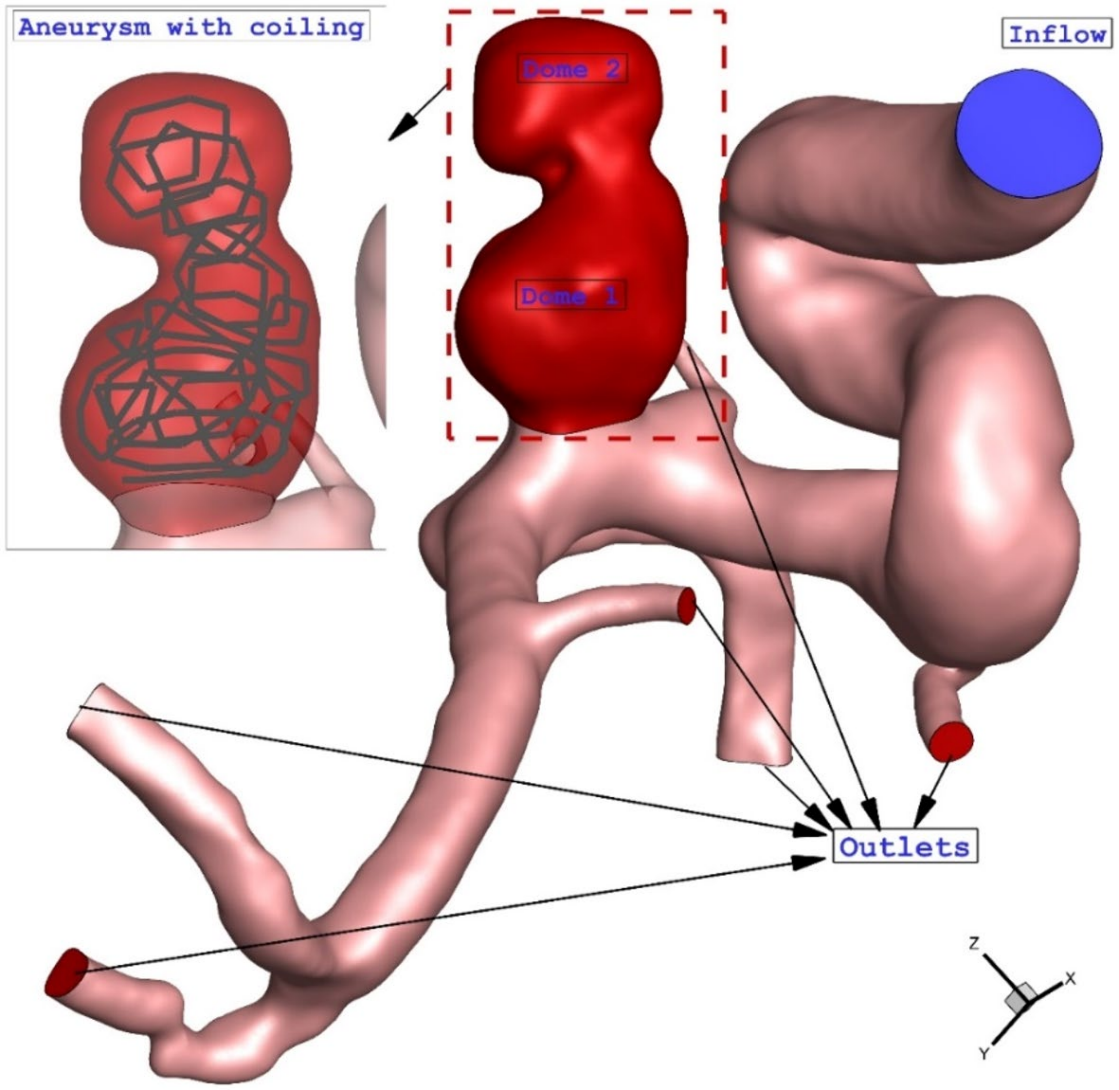
Schematic selected aneurysm.

Although several investigations have done to investigated blood hemodynamic within aneurysm, limited computational studies have presented hemodynamic details on aneurysm with double domes^27^. Besides, the influence of haematocrit factors on performance of coiling technique have been presented in limited works. In addition, one-way FSI model was not used for the modelling interaction of the blood with artery wall. Hence, this study tries to focus on the ICA aneurysm with double domes (Fig. 1) via one-way FSI model. The primary novelty of this work is related to the shape of selected aneurysm. Most of investigations have focused on simple aneurysm while this work present blood flow inside the aneurysm with double domes. In addition, in present work, real geometry of aneurysm is used for the simulations rather than simple arbitrary model. This study also investigated the impacts of blood haematocrit which is related to blood characteristics while it was not reported this effects on the previous work.

In our study, comprehensive CFD studies have been done investigate the impact of blood wave form on the hemodynamic characteristic of ICA aneurysms. Pressure distribution, WSS and OSI are compared in four main points to find critical region for the blood hemorrhage. Besides, the effects of the cyclic flow on maximum WSS are also presented in this research. The influence of blood viscosity and coiling technique on these high-risk regions are extensively explained.

## Material and methods

It is confirming that all methods were carried out in accordance with relevant guidelines and regulations. Besides, all experimental protocols were approved by of the Ca' Granda Niguarda Hospital and it is confirmed that informed consent was obtained from all subjects and/or their legal guardian(s).

**Figure 2:**
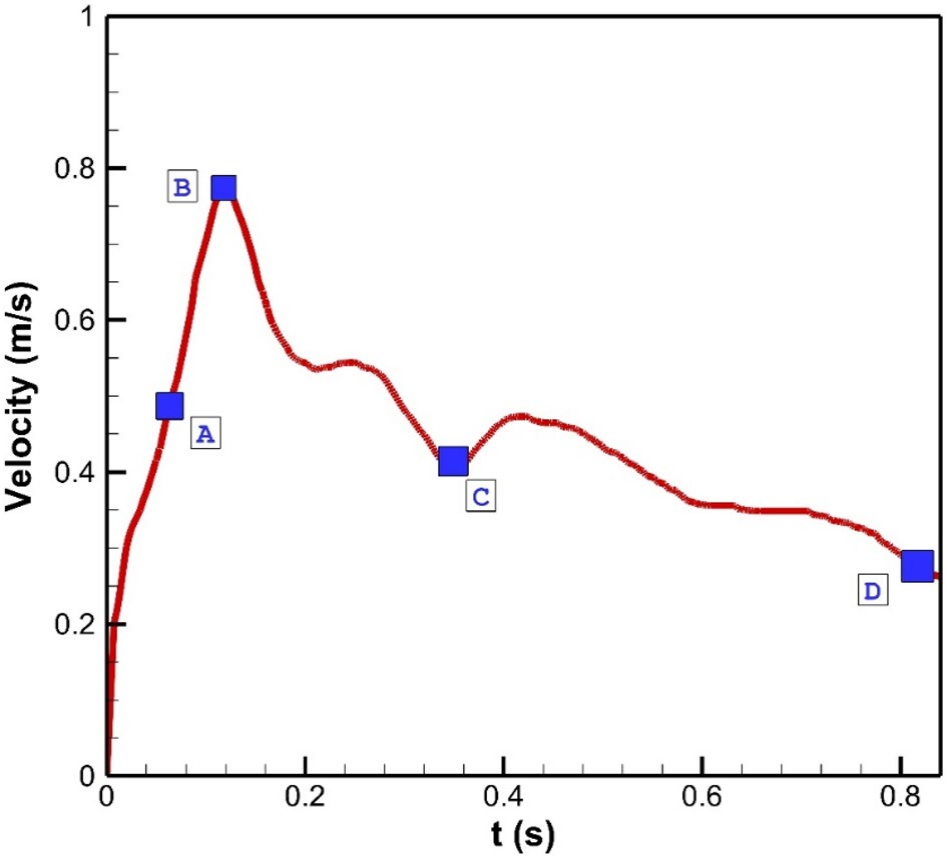
Inflow blood velocity profile^11^.

The simulation of the blood stream inside the model is done via solving the Navier–stokes equation with the laminar condition. This technique is extensively used in engineering problems^28–31^. The flow is assumed transient, non-Newtonian, and incompressible and a one-way FSI technique is applied for the modeling of the blood flow in chosen ICA model. In fact, the effects of the blood pressure on the aneurysm wall are considered. The real three-dimensional geometry of ICA is selected from a database of Emory University (aneurisk)^32^. The transitional profile of blood inflow is obtained from Ref.^33^ which is widely applied in previous works (Fig. 2). Three cycles of pulsatile flow at the inlet and pressure at the outlets were applied to ensure about the cyclic effects on our model is stable as demonstrated in Fig. 2. For the transitional study, four main points on the last cycle are chosen for quantitative comparison of the data and models^34^. In recent years, the computational fluid dynamics (CFD) method has been implemented in industries to model real engineering problems ^35–39^.

The Casson model is used for the calculation of the blood viscosity in our work. This work tries to reveal the effect of blood viscosity on the distribution of WSS and OSI inside the cerebral aneurysm: Therefore, two haematocrit values of 35% (female) and 45% (normal male) are studied. Haematocrit is related to red blood cells^27^. We used a correlation for calculation of the viscosity (Casson model) in relation with Hematocrit value as follow:

1 $$\mu =0.1\left({\left[\sqrt{\eta }+\sqrt{{\tau }_{\gamma }\left(\frac{1-{e}^{-m\left|\dot{\gamma }\right|}}{\left|\dot{\gamma }\right|}\right)}\right]}^{2}\right)and {\tau }_{\gamma }={\left(0.625H\right)}^{3}$$

Besides, the effect of coiling is also investigated in the present work by applying the porosity inside the sac of the aneurysm. To achieve this concept, two porosity values of 0.79 and 0.89 are considered to fulfil the role of the coiling technique in our selected aneurysm. OSI and WSS as two main factors for the evaluation of blood hemodynamic are calculated.

**Figure 3:**
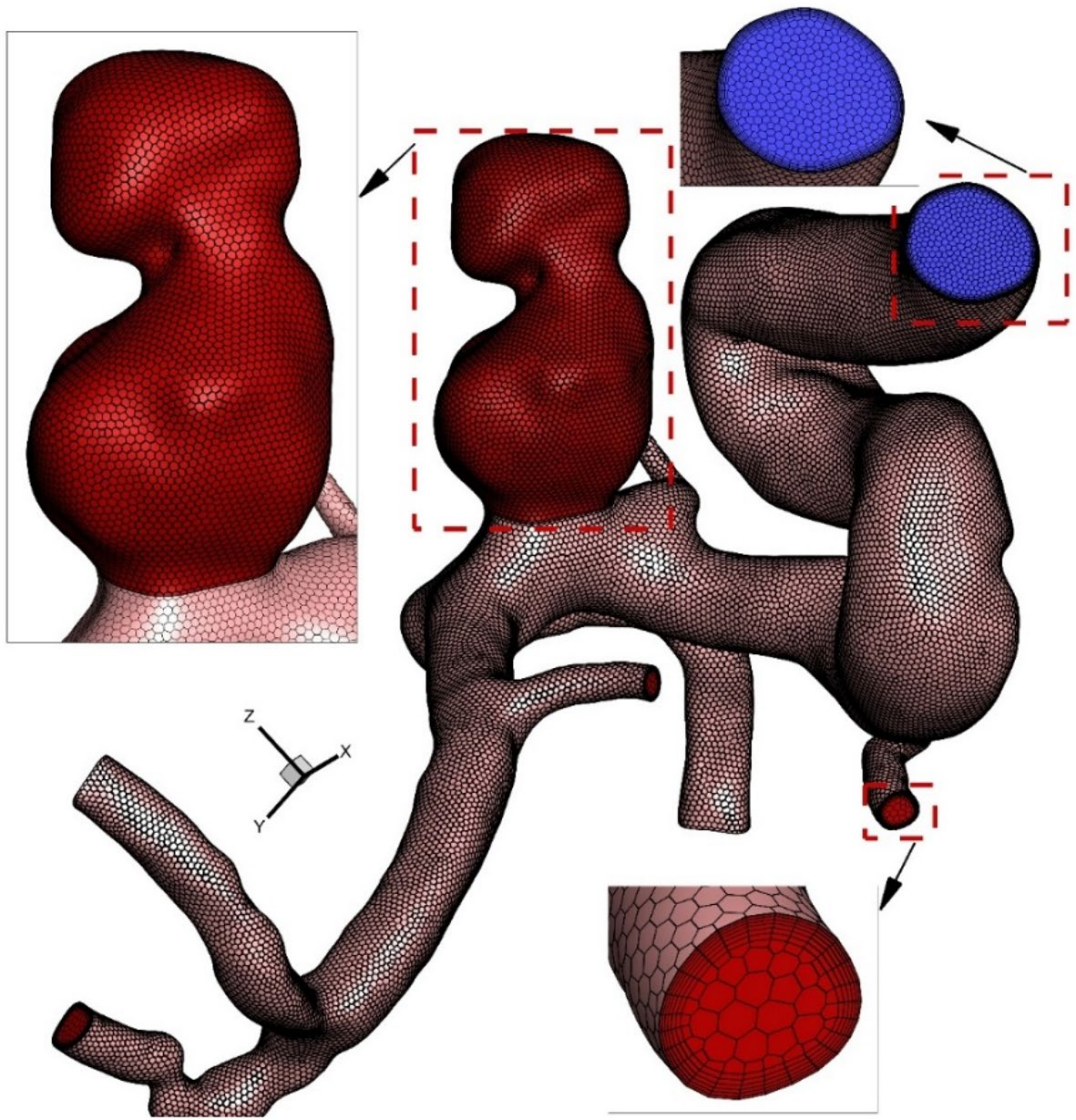
Generated grid.

Figure 3 illustrates the produced grid for our selected aneurysm. The selected model has 1.62 million cells that are high-resolution nearby the vessel wall. As demonstrated in Fig. 3, the grid size is reduced near the wall to disclose the main effects of the blood stream on our model. As noticed in the section view, the boundary layer is used for the grid production near the aneurysm wall due to importance of hemodynamic factors. In this research, hybrid grid is applied for reducing computational cost. As illustrated in the Fig. 3, structured hexagon grid is used near the vessel wall while polyhedron grid is applied in the centre of wall. This combination of grid helps us to reduce the number of the generated grid while the required precision near the wall is maintained. To ensure the precision of the grid, the mean value of the average velocity on the inlet of an aneurysm sac is compared for four different grids and the results are presented in Table 1. According to our comparison, the fine grid is a reasonable selection for our investigation.

**Table 1:** Details of used grids.

	Cells	Average blood velocity at inlet of sac (maximum acceleration)	Average blood velocity at inlet of sac (peak systolic)
Coarse	722,000	0.342	0.51
medium	1,120,000	0.361	0.54
fine	1,624,000	0.379	0.56
Very fine	2,064,000	0.382	0.561

## Results

**Figure 4:**
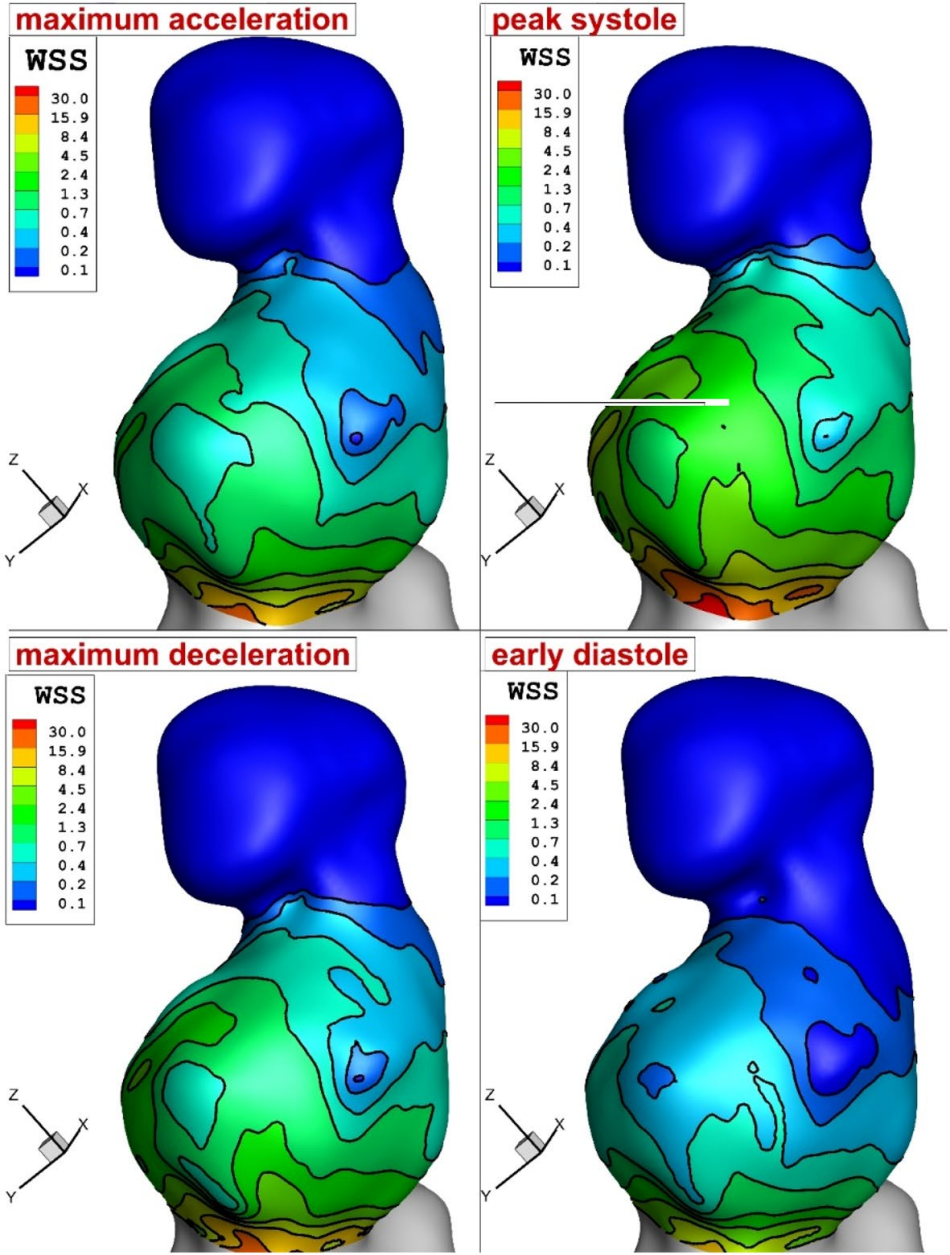
WSS distribution in different time instants.

In this work, we initially demonstrate the impacts of pulsatile blood flow on the main factors i.e. pressure, velocity, OSI, and WSS distribution on an aneurysm wall. Figure 4 illustrates the change of the WSS on the aneurysm wall in different time instants for models with porosity of 79% and HCT = 0.35 (female patient). Our results show that high WSS occurs at the neck of the aneurysm sac while the upper dome has less WSS.

**Figure 5:**
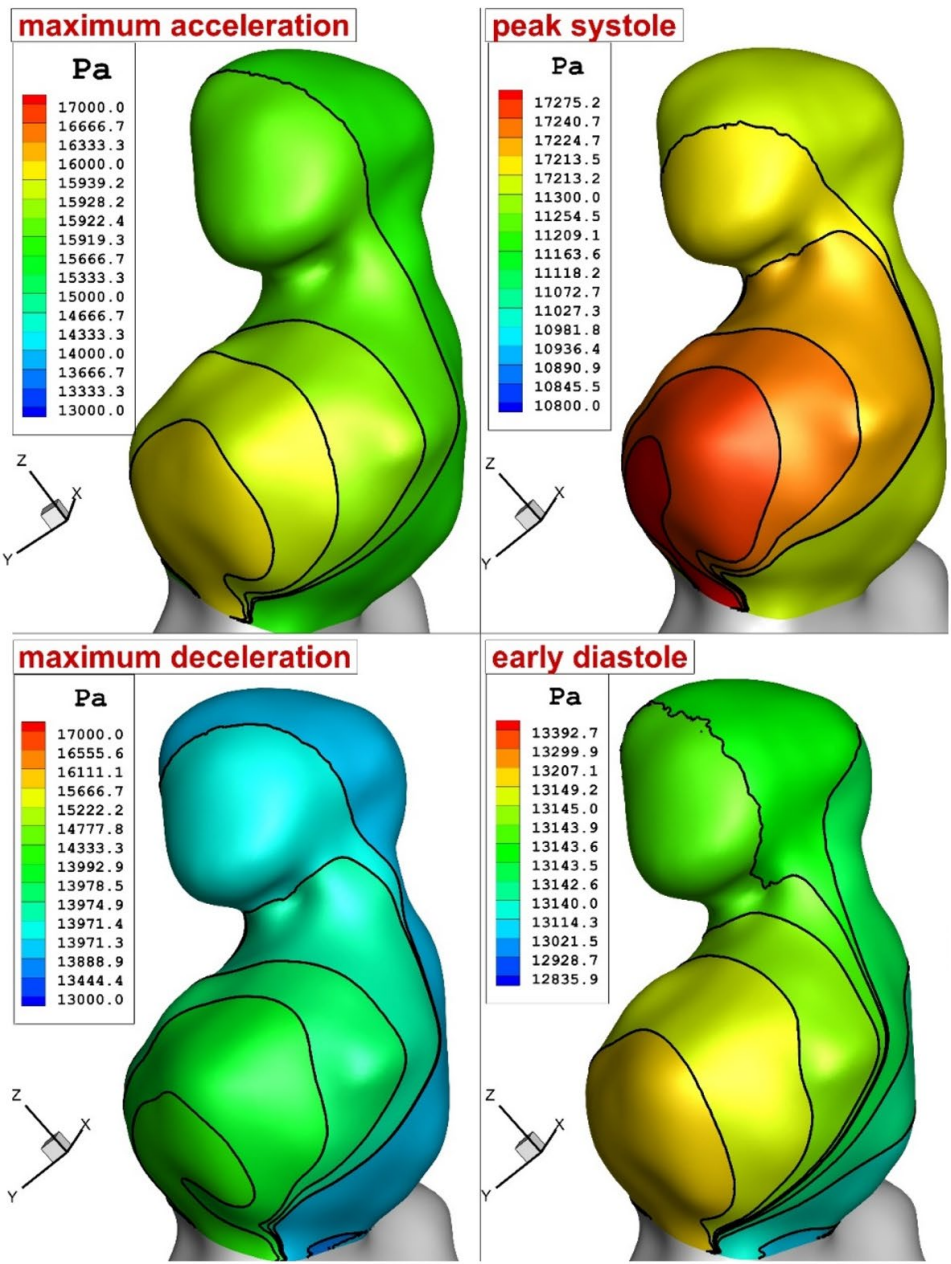
Wall pressure distribution in different time instants.

The pressure variation inside the sac offers valuable information about the impacts of blood stream on the formation and growth of the aneurysm (Fig. 5). As expected, the pressure of the down dome is more than the upper one and maximum pressure is noticed in the region with high curvature. Besides, the gradient of the pressure is maximum in peak systolic stage because of the high mass flow rate of the blood streaming inside the aneurysm.

**Figure 6:**
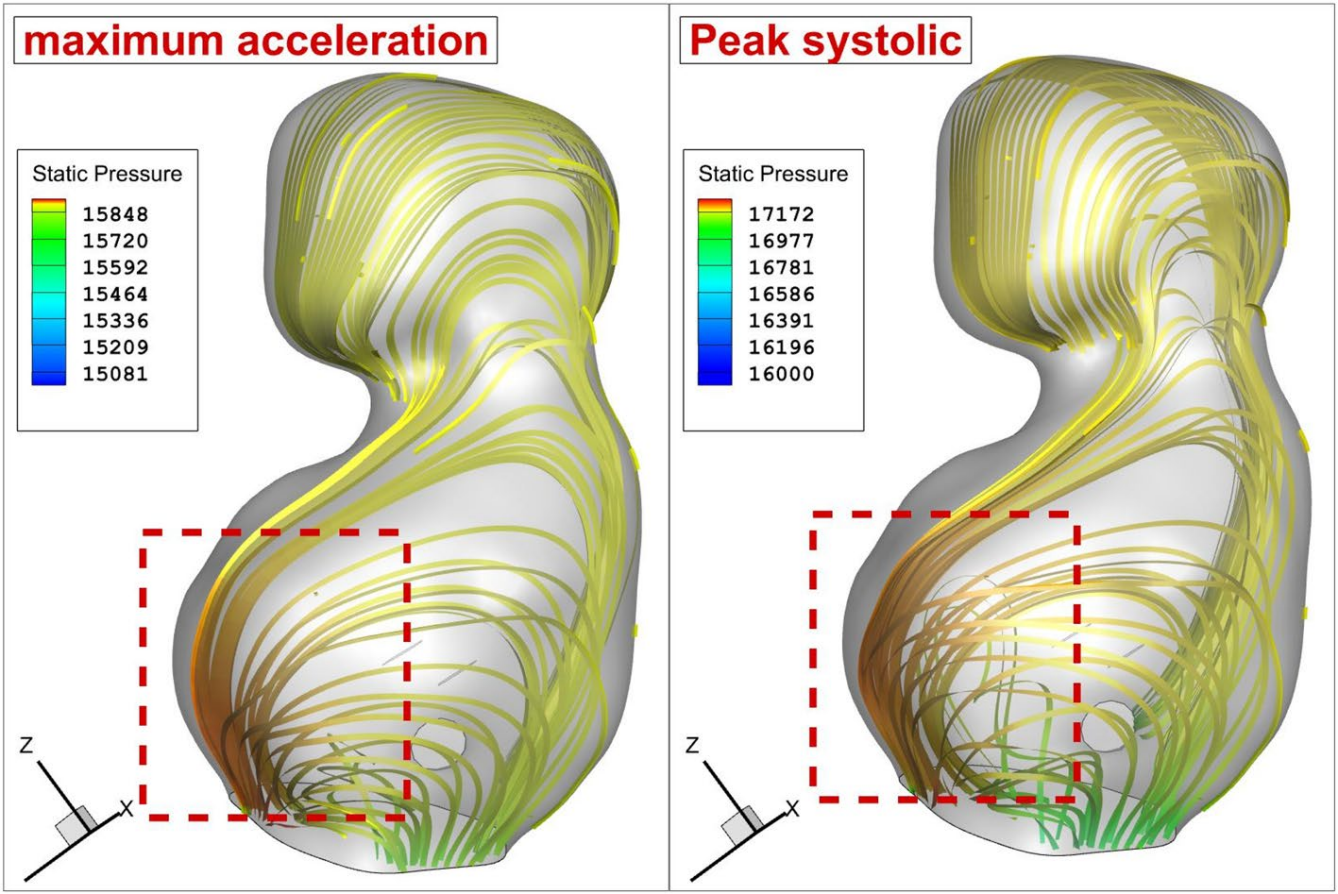
Blood flow with pressure value.

Figure 6 illustrates the flow impingement zone to disclose the potential association between the flow impingement and the pressure variation. As illustrated in the figure, pressure of the blood rises in the region near neck of the aneurysm. In fact, this is mainly due to the blood impingement on the aneurysm wall.

**Figure 7:**
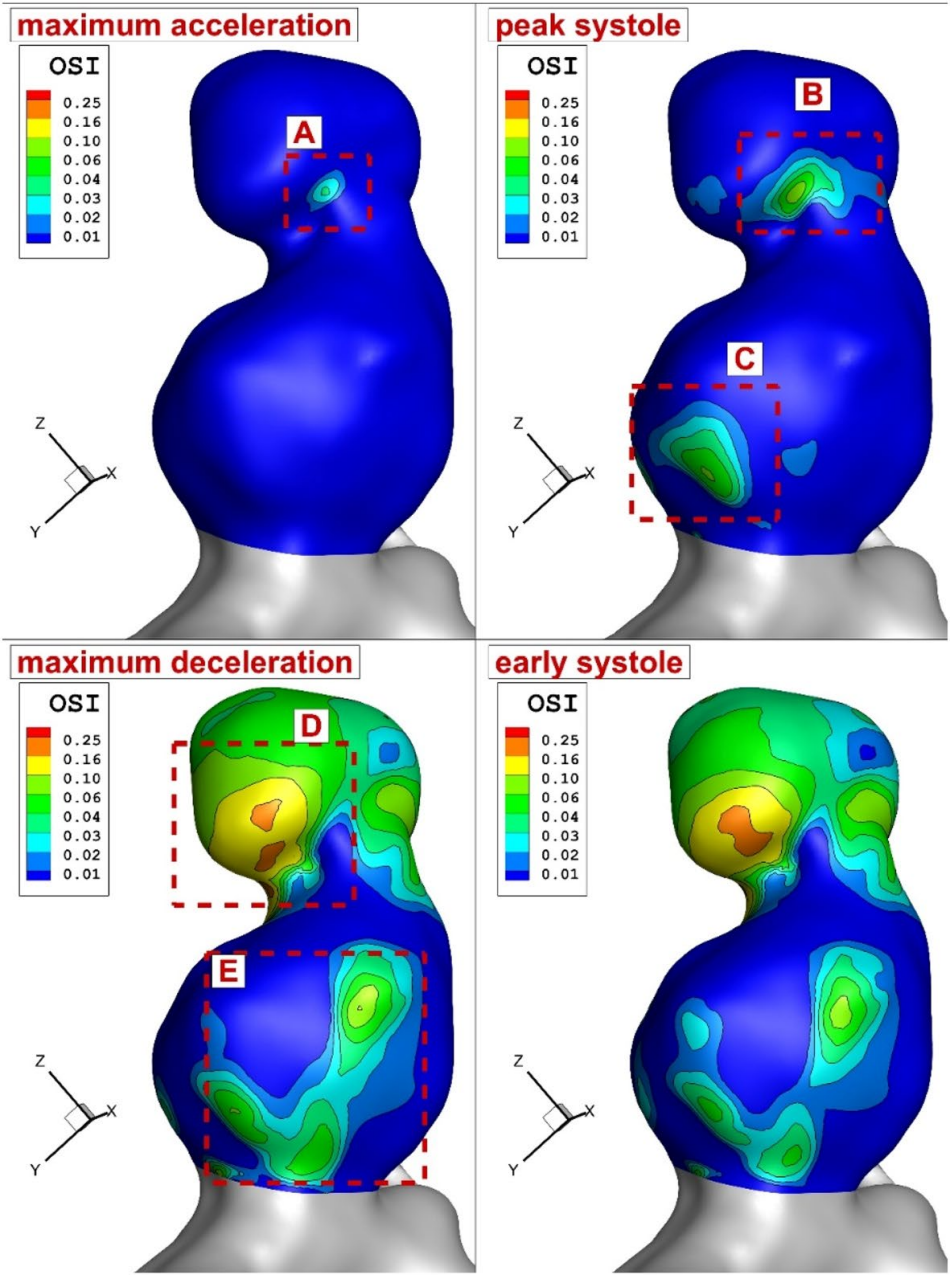
OSI distribution in different time instants.

**Figure 8:**
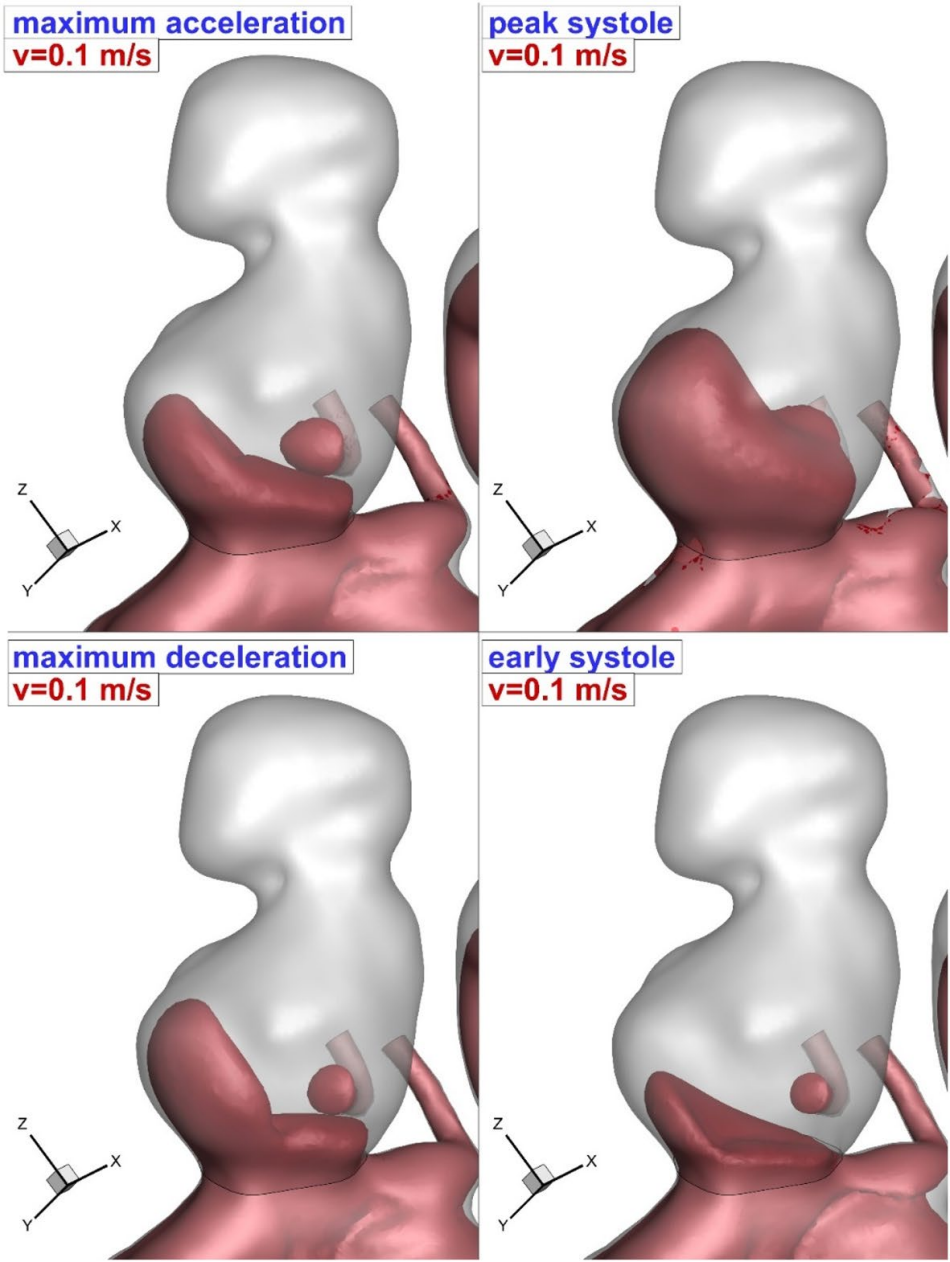
Blood contour in different time instants.

The comparison of the OSI indicates that the initial high OSI happens on the section between upper and lower dome (zone A) at maximum acceleration stage (Fig. 7). OSI is mainly indicates change of the wall shear stress. Since the velocity value is lower in upper dome, velocity gradient is higher and this results in higher OSI values in the upper dome. As blood incoming velocity is increased, the second section with high OSI is produced in the lower dome (section C) while the first one is extended. The maximum OSI value is noticed at maximum deceleration stage due to sharp change in the reduction of the blood velocity. In this stage, the upper dome is fully covered by high OSI value. Figure 8 illustrates the blood hemodynamic in different stages of the blood cycle. The variation of blood indicates the impact of blood velocity on the diffusion of the blood into the sac area.

**Figure 9:**
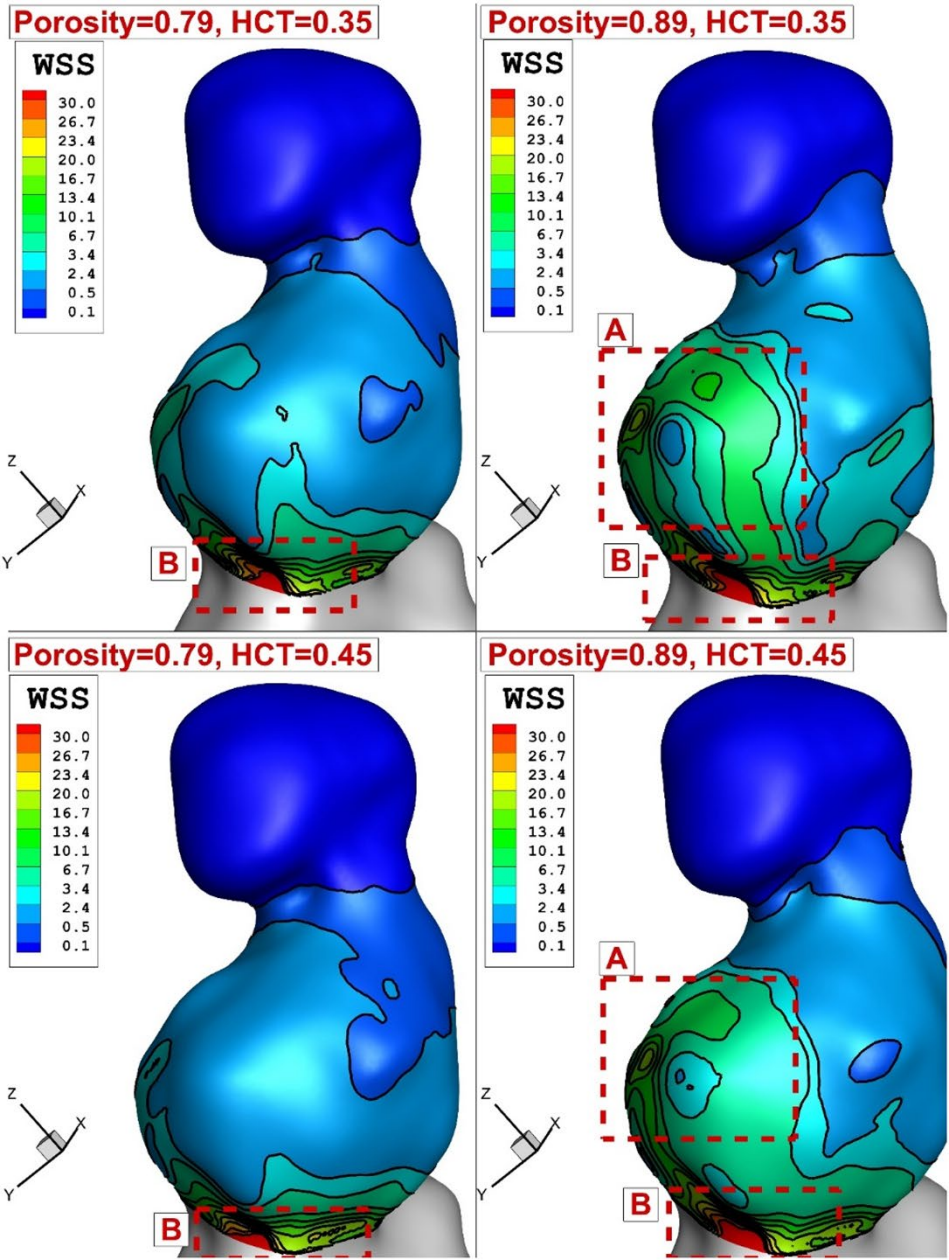
WSS distribution on aneurysm wall in different porosities and HCTs.

**Figure 10:**
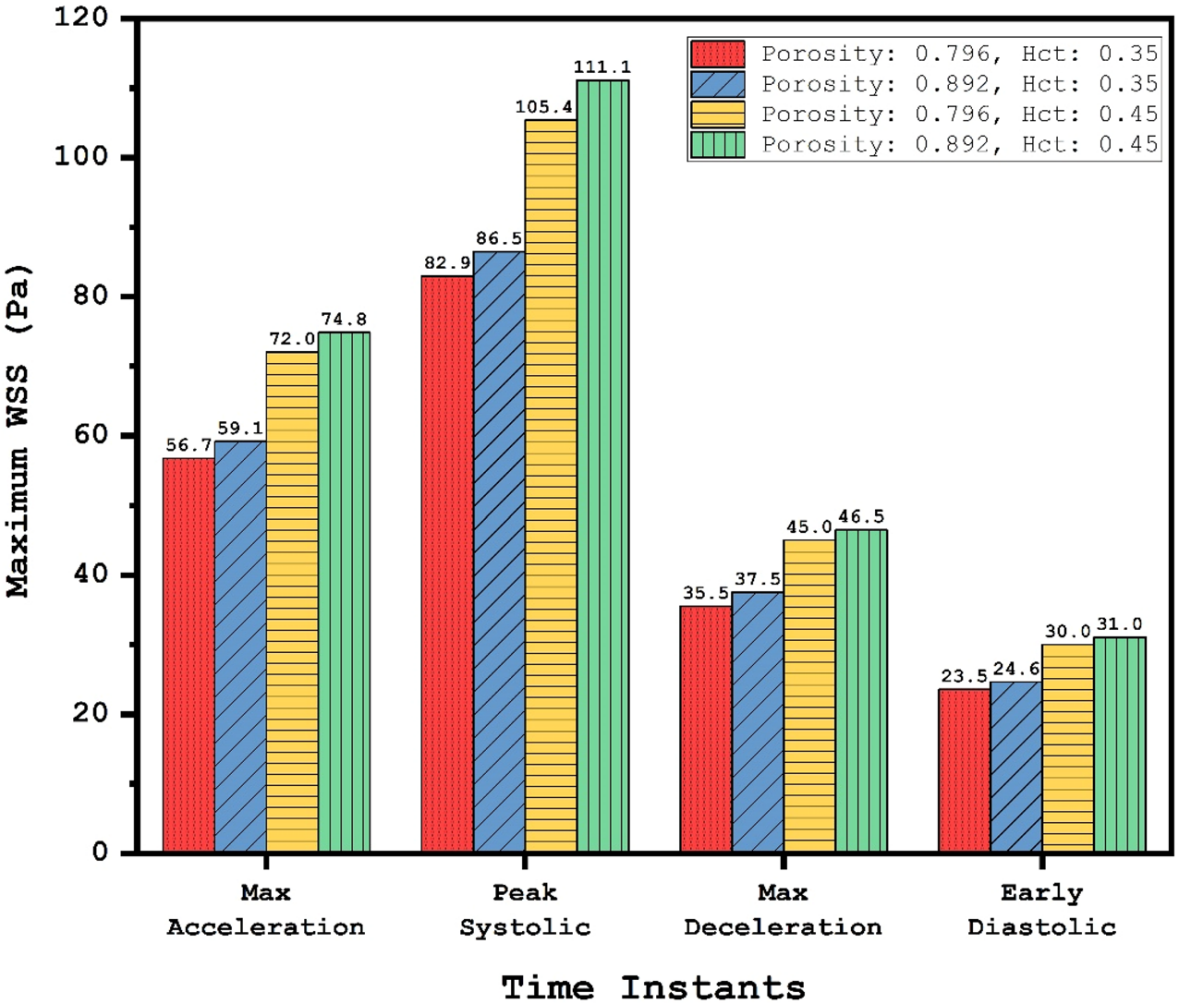
Effects of coiling (porosity) and patient sexuality (HCT) on the maximum WSS in different time instants.

The effect of the coiling porosity and blood HCT on the distribution of WSS are demonstrated in Fig. 9 at the systolic stage. Our results show that a reduction of the porosity (equivalent to an increase in permeability) substantially decreases the WSS on the wall of an aneurysm. Quantitative evaluation of maximum WSS for different stages is presented in Fig. 10. It is found that the HCT effect is noticeable (25% reduction in maximum WSS) in the two first stages (maximum acceleration and peak systolic). According to obtained results, a reduction of porosity from 0.89 to 0.79 would decrease maximum WSS by about 8% in both HCT conditions.

**Figure 11:**
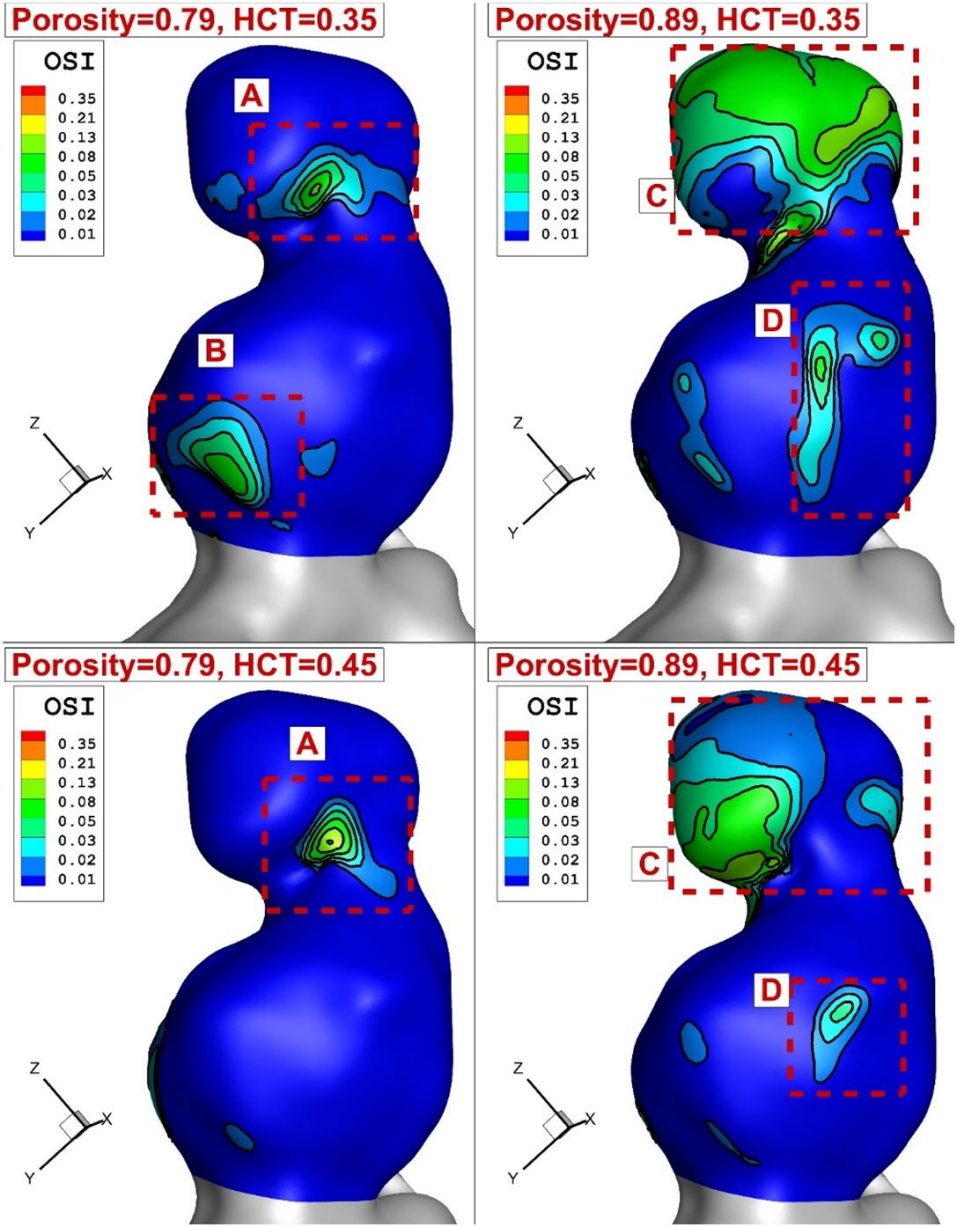
Change of OSI on the aneurysm wall in different HCTs and Coiling porosities.

Figure 11 illustrates the OSI change due to variation of the blood HCT and coiling porosity in peak systolic instant. As expected, the reduction of porosity considerably limited the high OSI area on the aneurysm wall, especially in the upper dome. Meanwhile, the regions with high OSI values are restricted more in high HCT (HCT = 0.45). This confirms the role of blood viscosity on the structure of the aneurysm.

**Figure 12:**
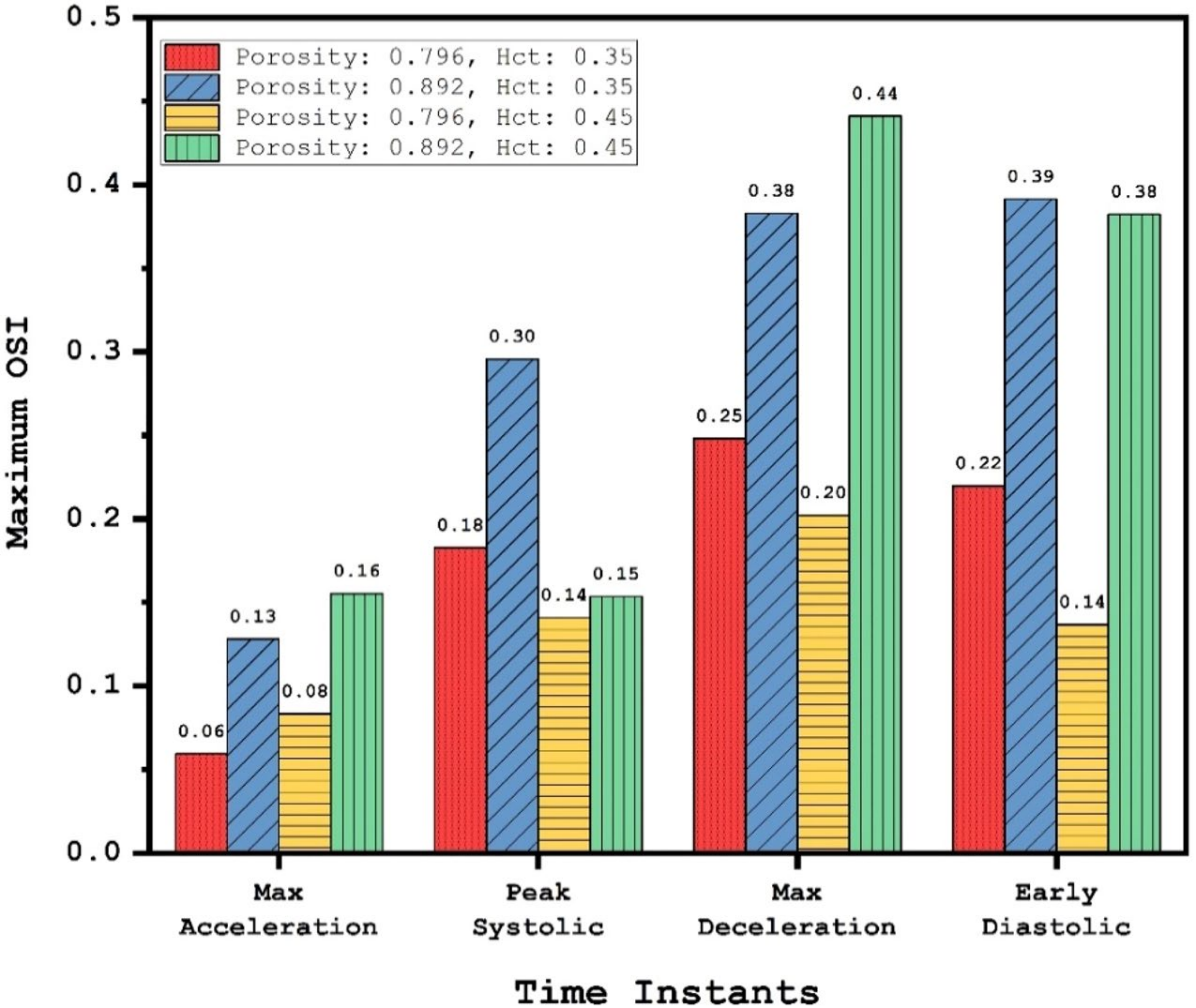
Comparison of Maximum OSI on the aneurysm wall in different HCTs and Coiling porosities.

A quantitative comparison of the maximum OSI for different instants is presented in Fig. 12. It is found that the effect of coiling porosity on the reduction of maximum OSI is more than 45% for the two last instants (maximum deceleration and early diastolic) when the porosity of the coiling is reduced from 0.89 to 0.79. However, HCT impacts are limited in these two stages.

**Figure 13:**
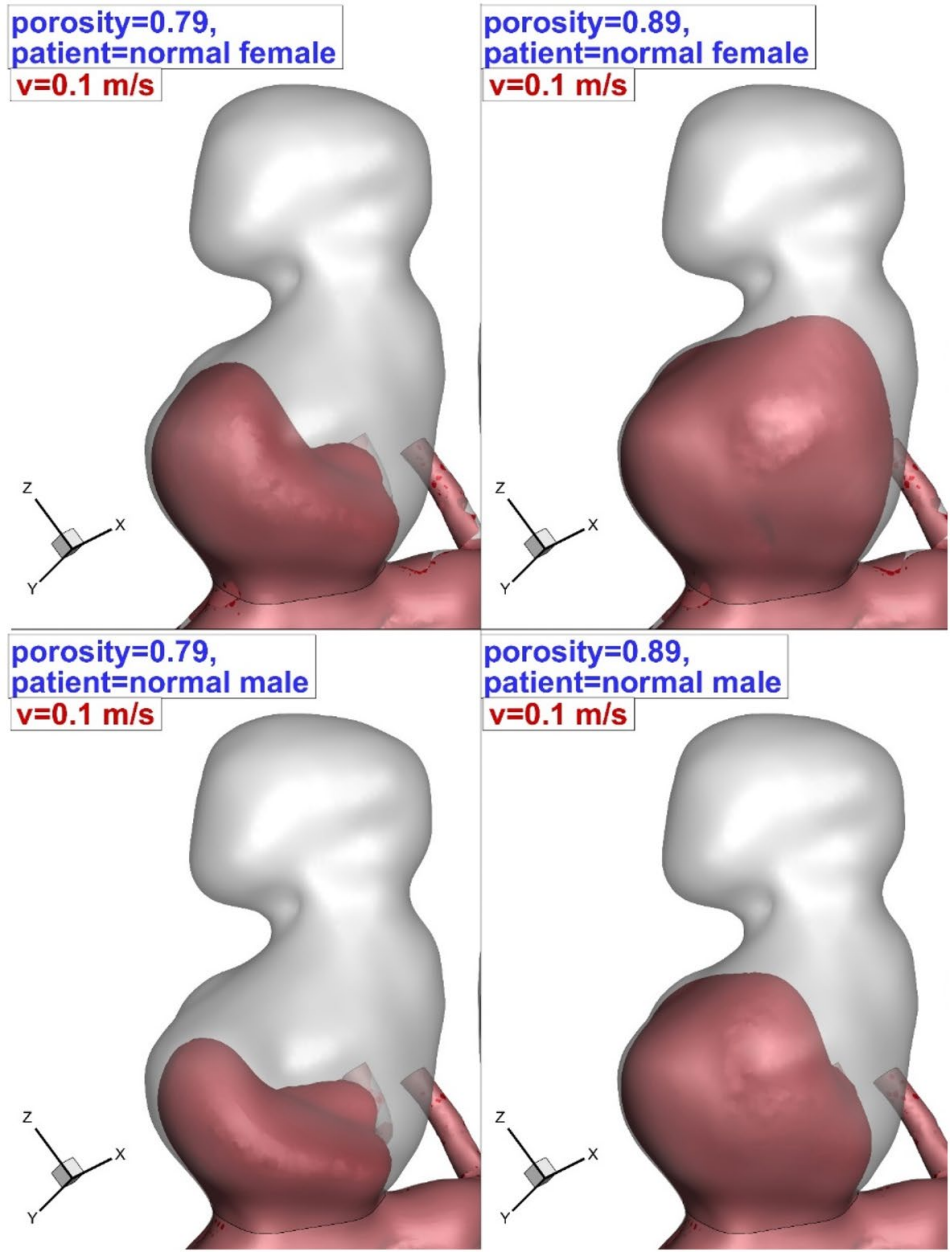
Blood hemodynamic (iso-surface of v = 0.1 m/s).

To observe the main impacts of the coiling, blood iso-velocity contour is demonstrated in Fig. 13. The feature of the blood is observed in diverse blood HCT and coiling porosities. This contour confirms that the blood interaction with the aneurysm wall is substantially limited with high permeability (equivalent to low porosity) of coiling. Besides, the comparison of the upper and lower domes indicates that the upper dome has less interaction with bloodstream than lower one.

**Figure 14:**
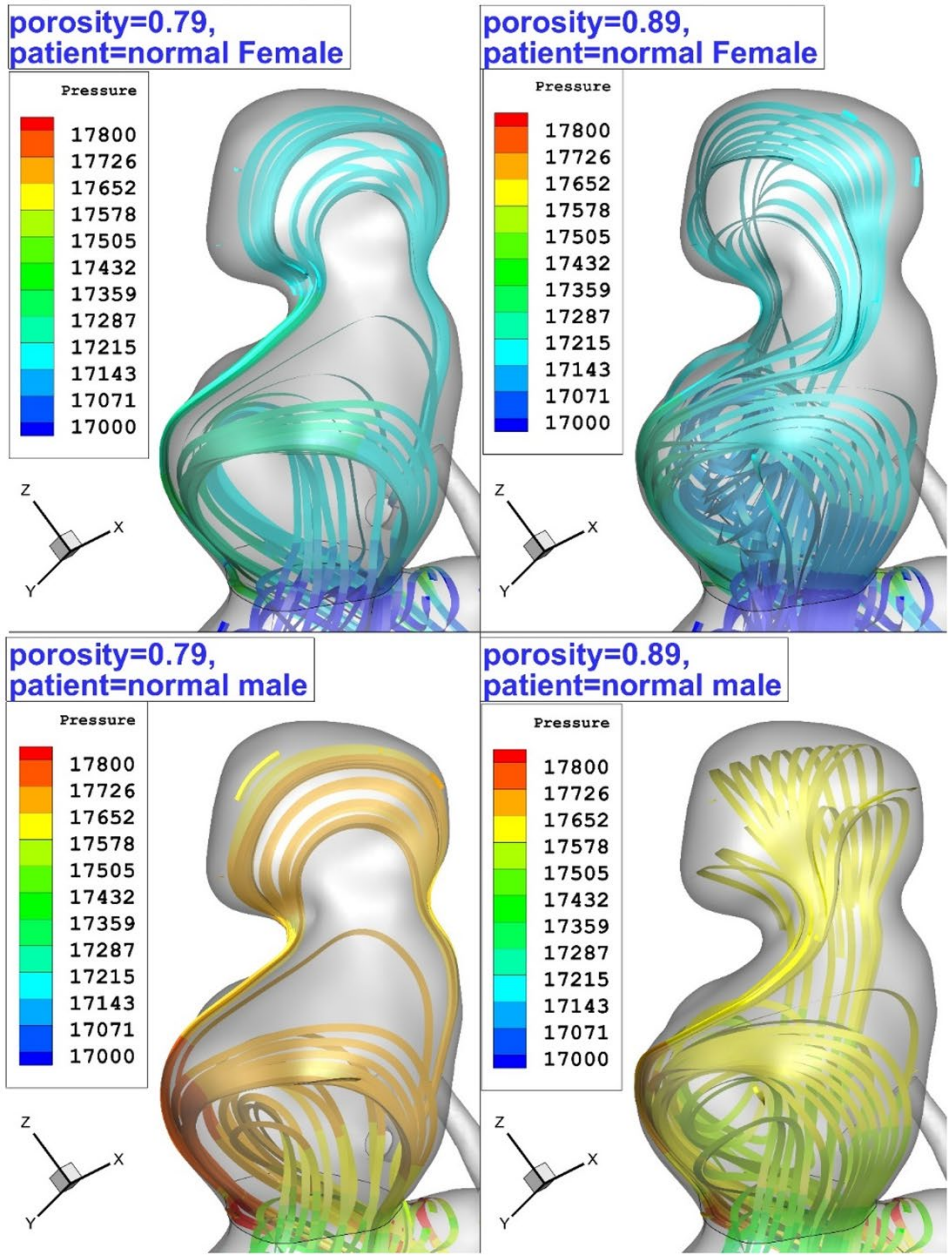
Blood stream patter inside the aneurysm in different HCTs and Coiling porosities.

The bloodstream analysis is also done to present a physical overview and find the main source of WSS on the aneurysm wall. Figure 14 illustrates the blood flow pattern inside the sac of the selected aneurysm. It is perceived that more portion of the bloodstream creeps on the aneurysm wall and this is highly visible in the lower dome. In a normal patient (HCT = 0.45), blood pressure is higher than normal female (HCT = 0.35).

**Figure 15:**
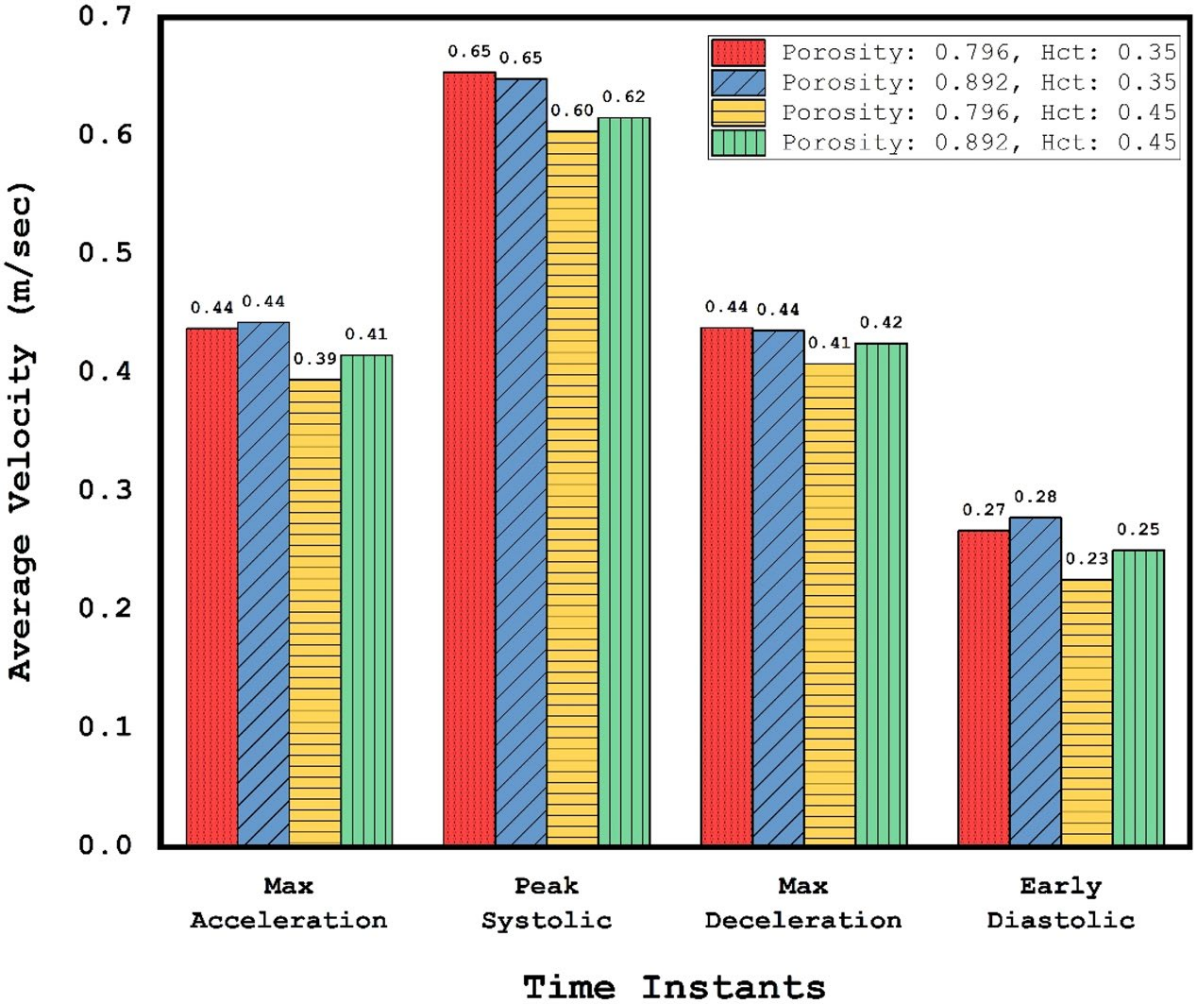
Comparison of the average velocity at inlet of sac for different HCTs and Coiling porosities.

Figure 15 demonstrates the average velocity at the inlet of the sac for different HCTs and coiling porosities. It is noticed that the effects of coiling porosity and blood HCT are not substantial in different stages. The main change in the value of the average velocity is related to the velocity of the blood stream at the inlet.

## Conclusion

The influence of the blood viscosity and coiling treatment on the hemodynamic characteristics of blood stream is fully investigated. CFD method is employed to study the wall shear distribution over the wall of the cerebral aneurysm to determine the high-risk regions that may rupture. The primary focus is on the evaluation of blood hemodynamics on the progress of the aneurysm. Our results show that in maximum acceleration and peak systolic stages, the HCT effect is noticeable (25% reduction in maximum WSS). According to obtained results, a reduction of porosity from 0.89 to 0.79 would decrease maximum WSS by about 8% in both HCT conditions.

## Data Availability

All data generated or analysed during this study are included in this published article.
